# PRT-Net: a progressive refinement transformer for dose prediction to guide ovarian transposition

**DOI:** 10.3389/fonc.2024.1372424

**Published:** 2024-05-16

**Authors:** Shunyao Luan, Yi Ding, Changchao Wei, Yi Huang, Zilong Yuan, Hong Quan, Chi Ma, Benpeng Zhu, Xudong Xue, Wei Wei, Xiao Wang

**Affiliations:** ^1^ The Institute of School of Integrated Circuits, Wuhan National Laboratory for Optoelectronics, Huazhong University of Science and Technology, Wuhan, China; ^2^ Department of Radiation Oncology, Hubei Cancer Hospital, TongJi Medical College, Huazhong University of Science and Technology, Wuhan, Hubei, China; ^3^ Key Laboratory of Artificial Micro and Nano-structures of Ministry of Education, Center for Theoretical Physics, School of Physics and Technology, Wuhan University, Wuhan, China; ^4^ Department of Radiation Oncology, Rutgers-Cancer Institute of New Jersey, Rutgers-Robert Wood Johnson Medical School, New Brunswick, NJ, United States

**Keywords:** artificial intelligence, deep learning, dose prediction, ovarian transposition, radiotherapy

## Abstract

**Introduction:**

Young cervical cancer patients who require ovarian transposition usually have their ovaries moved away from the pelvic radiotherapy (RT) field before radiotherapy. The dose of ovaries during radiotherapy is closely related to the location of the ovaries. To protect ovarian function and avoid ovarian dose exceeding the limits, a safe location of transposed ovary must be determined prior to surgery.

**Methods:**

For this purpose, we input the patient's preoperative CT into a neural network model to predict the dose distribution. Surgeons were able to quickly locate low-dose regions based on the dose distribution before surgery, thus determining the safe location of the transposed ovary. In this work, we proposed a new progressive refinement transformer model PRT-Net that can generate dose prediction at multiple scale resolutions in one forward propagation, and refine the dose prediction using prediction details from low to high resolution based on a deep supervision strategy. A multi-loss function fusion algorithm was also built to fit the prediction results under different loss dimensions. The clinical feasibility of the method was verified through an actual cases.

**Results and discussion:**

Therefore, using PRT-Net to predict the dose distribution by preoperative CT in cervical cancer patients can assist clinicians to perform ovarian transposition surgery and prevent patients' ovaries from exceeding the prescribed dose limit in postoperative radiotherapy.

## 1 Introduction

Cervical cancer is currently the most common cancer of the female reproductive system globally, with approximately 30%–40% of cervical cancers occurring in young women (1). Patients with early-stage cervical cancer who have high pathological risk factors for recurrence after radical hysterectomy need adjuvant radiotherapy or chemotherapy. The dose of conventional postoperative radiotherapy for cervical cancer is 4,500 cGy or greater, but the ovaries are sensitive to radiation. Some studies have shown that doses above 400 cGy can lead to permanent ovarian failure, early menopause, some menopausal syndromes, etc. (2). As a result, young patients often undergo ovarian transposition during a radical hysterectomy to move their ovaries out of the pelvic region that is potentially the field of radiation, which can significantly reduce the dose to the ovaries. However, current data show that only approximately 50% of transposed ovaries retain their function, even if the ovaries have been removed from the potential radiation field (3–5). The ovarian survival rate still needs to be improved. Studies have shown that the maintenance of ovarian endocrine function after radiotherapy in cervical cancer patients is directly related to ovarian dose (6, 7), which is closely associated with the location of the transposed ovaries. Therefore, during ovarian transposition, it is particularly important to determine the appropriate location for ovarian placement and ensure that the dose of the ovaries in postoperative adjuvant radiotherapy is below safe limits.

Current research on the location of the transposed ovary is mainly based on statistical methods (8–10). By analyzing data from 150 patients who had undergone ovarian transposition and received postoperative radiotherapy, LV et al. (8) plotted the operating characteristic curve (ROC) of ovarian position *versus* the dose received and concluded that moving the ovaries above 1.12 cm in the iliac crest plane enabled the dose to be controlled below the safety limit. J Toman et al. (9) monitored the ovarian endocrine function following pelvic external beam radiation with the ovaries at various transposition locations and discovered that the ovaries were safe beyond 2.5 cm of the radiation field edge. Nevertheless, each patient’s anatomy and pathology staging affect the size of the target area in radiation. Even if the ovaries are all located 2.5 cm from the radiation field edge for various people, the radiation dose may differ significantly. As a result, the findings from earlier studies were not patient-specific and might not be appropriate for all individuals.

Researchers have recently utilized deep learning to predict the dose distribution for several cancer types, including prostate, rectal, and cervical cancers (11–13). The U-net and several U-net-like models are typically the foundation of current experiments employing deep learning for the dose prediction (14–16). However, it has been documented that due to the physical characteristics of volumetric modulated arc therapy (VMAT) dose, traditional U-Net models often do not predict the VMAT dose distribution well, especially in low dose regions (17, 18). To improve the ability of neural network models to predict VMAT dose distribution, we propose a novel progressive refinement attention model with deep supervised strategy and weighting self-attention architecture, which can improve the generalization and robustness of the model. Then, the model is applied to the prediction for the dose distribution. The predicted dose distribution can be visualized on preoperative computed tomography (CT), which then can be used to determine the location of the ovary during ovarian transposition surgery so that the dose of the ovary in postoperative radiotherapy is below the safety limit. By using a neural network model that exploits the characteristic relationship between patient anatomy and dose distribution, the dose distribution can be predicted more accurately based on the unique anatomy of each patient, and thus the appropriate transposed ovarian location can be predicted.

## 2 Materials and methods

**Figure 1:**
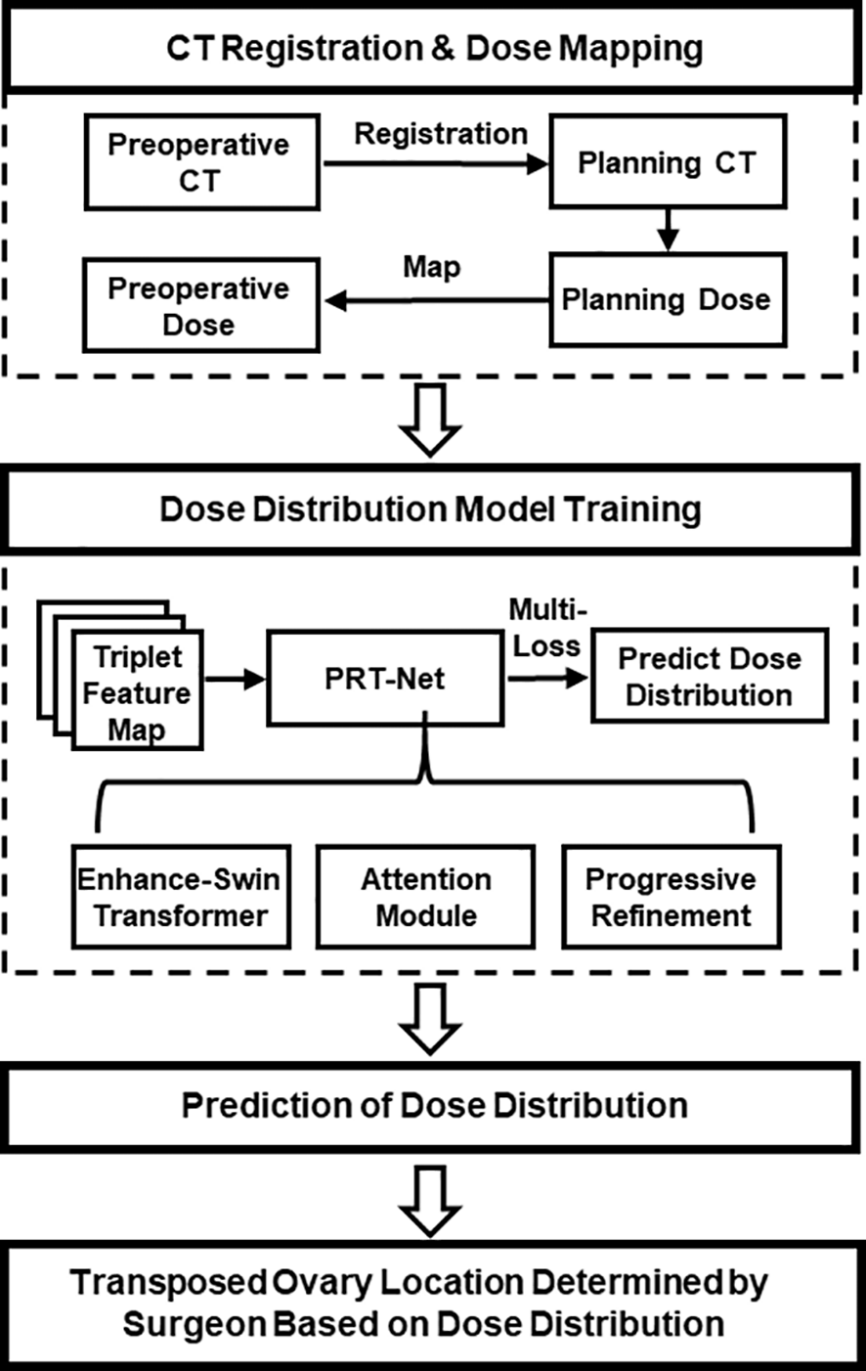
Overall workflow of the study.

In this study, a new progressive refinement attention model, PRT-Net, was used to predict the dose distribution. The preoperative CT slices, organs at risk (OARs), and PTVs were used as the input of the model. The dose distribution of the planning CT was mapped to the preoperative CT and used as the output of the model. Note that the planning CT is the CT taken after ovarian transposition, namely, postoperative CT. This output of the model combining the dose distribution with preoperative CT will provide a low dose region that will be the range for the ovarian transposition. The surgeons can then determine the safe location of ovary prior to surgery based on clinical knowledge within this range. A flow chart showing the process of this study is demonstrated in **Figure 1**.

### 2.1 Patients and treatment planning

In this study, the clinical data of 104 patients (69 cases were randomly selected as training data, 22 as validation data, and the remaining 13 as test data) with cervical cancer who received postoperative radiotherapy in Hubei Cancer Hospital, Wuhan, China, were collected. Prior to radical hysterectomy, preoperative CT was obtained using SOMATOM Definition AS+ (Siemens, Berlin, Germany), with a slice thickness of 5 mm. The patients were immobilized in the supine position by vacuum cushions. Planning CTs were acquired with a Brilliance CT (Big Bore, Philips, Cleveland, OH, USA), and the slice thickness was also 5 mm.

The clinical target volume (CTV) and OARs including bladder, rectum, small intestine, bilateral femoral heads, marrow, and spinal cord were delineated by an attending oncologist. The prescription was 45 Gy in 25 fractions (1.8 Gy per fraction) to the planning target volume (PTV) generated with 0.5 cm uniform expansion from the CTV. All VMAT plans are using two full arcs with 6-MV energy, designed in the Monaco treatment planning system (TPS) (version 5.11.01, Elekta, Stockholm, Sweden) with the Monte Carlo algorithm for an Infinity accelerator (Elekta, Stockholm, Sweden).

According to ICRU (International Commission on Radiation Units and Measurements) Report 83, D95% of the PTV is greater than the prescribed dose, D2% is less than 110% of the prescribed dose, and D98% is greater than 95% of the prescribed dose. For the evaluation of OAR for cervical cancer, reference was made to the RTOG (Radiation Therapy Oncology Group) Report 0418 and the actual situation of our institution. The corresponding evaluation criteria are as follows: V30<50%, V40<40%, V45<35% for bladder, V30<60%, V40<55% for rectum, V30<15% for femur head, and Dmax<45 Gy for spinal cord.

### 2.2 Dose alignment

The dose from the planning CT needs to be mapped to the preoperative CT in order to show the dose distribution on the preoperative CT, allowing surgeons to quickly determine the safe location of the transposed ovarian before the surgery. The image processing software MIM (version 6.8.7, MIM Software Inc., USA) was used to achieve image registration between preoperative and planning CT of the same patient using rigid alignment. Considering that the uterus, lymph nodes, and other structures are removed during radical hysterectomy for cervical cancer, the OARs will be greatly deformed between the preoperative CT and the planning CT. Therefore, the contours of targets and OARs of the pre-operative CT of each patient were manually revised by the radiation oncologist.

### 2.3 Data preprocessing

Raw CT pixel values were converted to Hounsfield units (HU) on a scale between -1,000 and +400. Each OAR and PTV contours were filled with 0 (background) and 1 (foreground) to transform into the binary mask. The PTV, bladder, femoral head left, femoral head right, kidney left, kidney right, marrow, rectum, spinal cord, and body were chosen as the relevant structures for VMAT dose prediction. The dose distribution was resampled to a voxel size of 2.5 × 2.5 × 5.0 mm by using single linear interpolation. In addition, the original image feature matrix was resampled to 512 × 512 pixels using the zero filling method.

To allow our neural network to learn the volumetric features, training was performed using image triplets input (19), which combines three consecutive 2D CT slices and their corresponding binary segmentation masks. Each 2D CT slice and a binary segmentation mask are combined into a superimposed feature map with 11 channels, denoted as $x\in R^{11\times 512\times 512}$ , then concatenated with three sets of spatially continuous superposition feature maps together along the channel dimension to form a final triplet feature map with 33 channels, denoted as $x\in R^{33\times 512\times 512}$ .

### 2.4 Quantitative dose prediction evaluation

To quantitatively evaluate the accuracy of the network model, we compared the dose prediction results of PRT-Net with three other neural network models [U-Net (20), U-net++ (21), and DeepLab-V3-plus (22)]. The evaluation metrics include mean absolute error (MAE), mean squared error (MSE), root mean square error (RMSE), absolute differences of dosimetric parameters between the real and predicted dose in the OARs and PTV, the dose–volume histogram (DVH), gamma index, and the isodose volume DSC, including the 4-, 10-, 15-, and 20-Gy isodose regions. All evaluation indexes are based on body contour as a mask, with the following formula:


$$\left\{[Neural\;Network\;(input\;feature\;map)]\;*(body\;contour),\;(ground\;truth)*\left(body\;contour\right)\right\}$$

Gamma index passing criteria was 3%/2 mm, and only the dose exceeding 10% of the maximum dose was calculated. In addition, areas outside the body were not included in the gamma index calculation.

The DSC of the isodose volume was evaluated in the 3D dose distribution. The DSC calculates the overlapping results of two different volumes based on Equation 1:


(1) $$DSC=\frac{2\left|A\cap B\right|}{\left|A\right|+\left|B\right|}$$

where *A* is the real isodose volume and *B* is the predicted isodose volume.

### 2.5 Implementation details

For training neural networks, Adam with a weight decay of 0.0001 was utilized to optimize network parameters, with the initial learning rate set to 0.0001. The batch size was set to 16, and the epoch size was set to 100. The neural network using PyTorch was implemented, and experiments were performed on a small NVIDIA RTX3090Ti workstation equipped with 24 GB of RAM.

## 3 Network architecture

**Figure 2:**
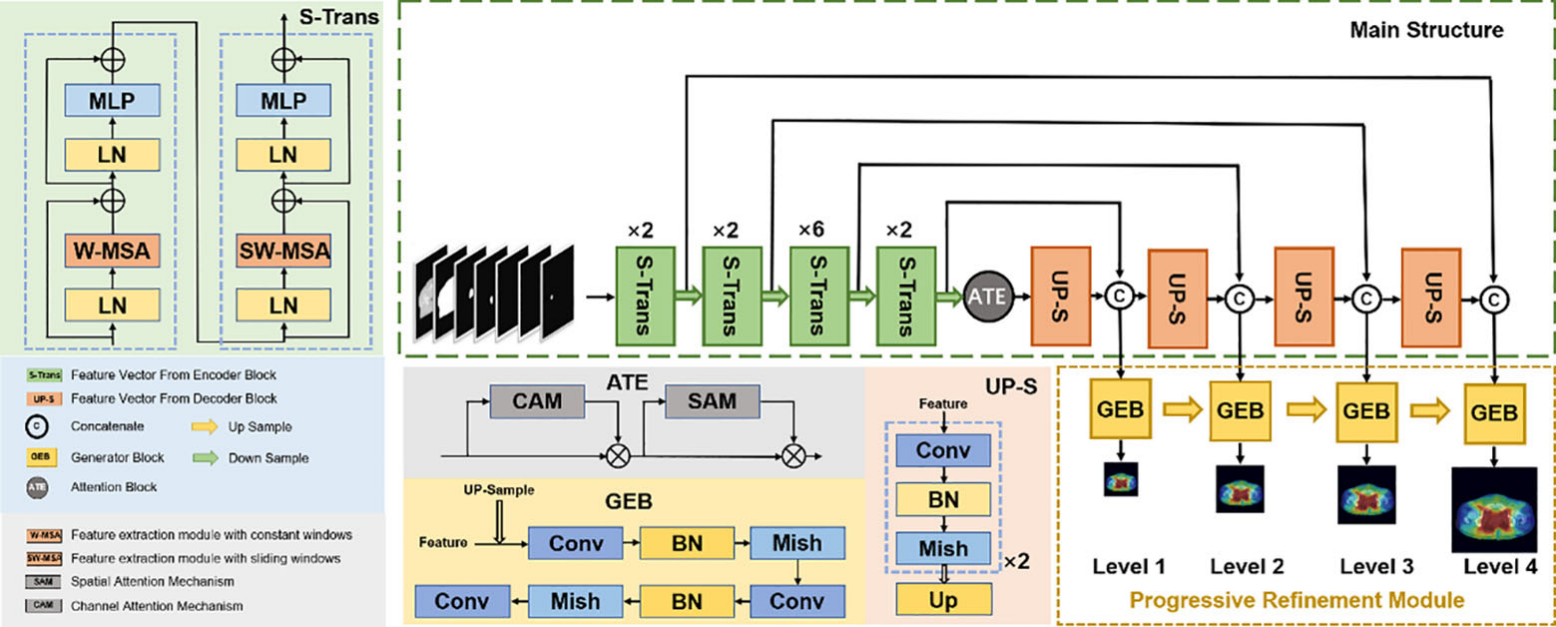
Schematic diagram of the proposed PRT-Net architecture.

We proposed a new progressive refinement attention model, Swin-Refinement-Attention (PRT-Net), based on the Swin-Transformer architecture (23), as shown in **Figure 2**. The model uses an efficient encoding module to extract superimposed feature map information, an attention module (24) to assign feature weights over space and channels and a decoder to gradually generate dose distribution from low- to high-scale resolution.

### 3.1 Encoding module

Considering that the traditional convolutional neural network (CNN) encoding architecture is unable to obtain global information at local locations and that the receptive field mechanism is prone to lose detailed semantic features, we adopted an encoding architecture based on the transformer, as shown in **Figure 2**. The encoding module consists of four enhanced Swin-Transformer encoders, each containing two mutually independent feature extraction modules (the constant window and the shifted window). Before feeding the input features into the enhanced Swin-Transformer layer, several pre-processing operations need to be performed on the raw feature maps. First, the original features $x\in R^{C\times H\times W}$ were split into N $\left(N=\frac{H}{P}\times \frac{W}{P}\right)$ tokenization $x\in R^{C\times P\times P}$ of the same size by the window splitting algorithm, where *P* is the patch size and *C* is the channel dimension. Then, to match the input of the enhanced Swin-Transformer layer, the linear projection function was used to convert the three-dimensional image patches into a two-dimensional embedding sequence $x_{e}\in R^{N\times c_{e}}$ , where *C_e_* represents the dimensionality of each embedding sequence. Finally, the generated embedding sequences were added with the prior position parameters to acquire the output sequence $x_{p}\in R^{N\times c_{e}}$ , which was fed directly into the enhanced Swin-Transformer layer.

Distinct from the traditional Swin-Transformer algorithm with a multi-headed self-attention mechanism (25), we adopted the multi-head-enhanced self-attention (enhanced multi-headed self-attention mechanism) architecture. First, three learnable matrixes (the query matrix $W^{Q}\in R^{C_{e}\times d_{k}}$ , the key matrix $W^{K}\in R^{C_{e}\times d_{k}}$ and the value matrix $W^{V}\in R^{C_{e}\times d_{v}}$ , where $d_{k}=d_{v}=\frac{C_{e}}{h},\;h$ represents an independent self-attention layer) with the output of the previous enhanced Swin-Transformer layer or input layer were used to calculate three sequence vectors (the query vector Q, the key vector K, and the value vector V), as shown in Equation 2. Then, the learnable weights matrix $W_{\alpha }\in R^{d_{k}\times N}$ was utilized to perform an enhanced self-attentive calculation on the query vector and the key vector to obtain the attention score, which was passed through the softmax activation function to acquire the normalized score, and then multiplied by each value vector to get the output vector $SA(x^{l})$ , as shown in Equation 3.


(2) $$Q=x^{l}W^{Q},K=x^{l}W^{K},V=x^{l}W^{V}$$

(3) $$SA(x^{l})=\text{softmax}\{\frac{[\tanh(Q+K)]\cdot W_{\alpha }}{\sqrt{d_{k}}}\}\cdot V$$

Multiple head cascades $W-MSA\left(x^{l}\right)$ for calculating the self-attention scores in different subspaces were generated to capture the correlation between different subspaces of the sequence, as shown in Equation 4, W−MSA denotes the enhanced self-attention algorithm with constant window, and then $x^{l+1}$ was obtained by summing with the original high-dimensional spatial features using the residual mechanism, as shown in Equation 5, with *LN* denoting layer normalization. Ultimately, the output of the constant window-based feature extraction module can be obtained by Equation 6; MLP denotes multi-layer perceptron.


(4) $$W-MSA\left(x^{l}\right)=\text{ Concat }\left[SA{\left(x^{l}\right)}_{1}....SA{\left(x^{l}\right)}_{h}\right]$$

(5) $$x^{l+1}=W-MSA\left[LN(x^{l})\right]+x^{l}$$

(6) $${\hat{x}}^{l+1}=x^{l+1}+\text{MLP}\left[LN(x^{l+1})\right]$$

The shift window feature extraction module SW−MSA was obtained by adding the window shift algorithm based on the W−MSA module. The cross-window connection introduced in SW−MSA while ensuring efficient computation of non-overlapping windows can fuse the feature information extracted from different windows and improve the model ability. The SW−MSA module employed window configurations distinguished from the regular window division of the W−MSA module, and SW−MSA module adopted shifting $\left[\frac{P}{2},\frac{P}{2}\right]$ pixels relative to the top left pixel point as the new window. Ultimately, the output of the SW−MSA module can be obtained by Equation 7.


(7) $$x^{l+2}=SW-MSA\left[LN({\hat{x}}^{l+1})\right]+{\hat{x}}^{l+1}+\text{MLP}\left[LN(SW-MSA\left[LN({\hat{x}}^{l+1})\right]+{\hat{x}}^{l+1})\right]$$

### 3.2 Attention module

To further improve the feature representation, we employed a lightweight convolutional attention operator that inferred attentional concentration regions along two specific and mutually independent dimensions and adaptively optimized local features by applying different attention scores for channel information and spatial information, respectively. The channel attention weight operator is shown in Equation 8. The channel weight operator was multiplied with the input feature map $x^{l+2}$ to obtain the channel enhancement feature map ${\hat{x}}^{l+2}$ , as shown in Equation 9. The spatial attention weight operator is shown in Equation 10. The spatial weight operator was multiplied with the channel-enhanced feature map ${\hat{x}}^{l+2}$ to obtain the channel–space enhanced feature map $x^{l+3}$ , as shown in Equation 11. Finally, the channel–space enhanced feature map $x^{l+3}$ was added to the input feature map $x^{l+2}$ to obtain the output feature map ${\hat{x}}^{l+3}$ of the attention module, as shown in Equation 12. Due to its smaller and lighter architecture, the convolutional attention operator can be seamlessly integrated into the network architecture while ignoring its cost to perform end-to-end training together with the neural network.


(8) $$M_{c}(x^{l+2})\;=\sigma (MLP(\text{Avg}P{\text{ool}}^{C\times 1\times 1}(x^{l+2}))+MLP({\text{MaxPool}}^{C\times 1\times 1}(x^{l+2})))$$

(9) $${\hat{x}}^{l+2}=M_{c}(x^{l+2})⊗x^{l+2}$$

(10) $$M_{s}({\hat{x}}^{l+2})=\sigma (C\text{on}v^{7\times 7}([\text{Avg}{\text{Pool}}^{1\times H\times W}({\hat{x}}^{l+2});{\text{MaxPool}}^{1\times H\times W}({\hat{x}}^{l+2})]))$$

(11) $$x^{l+3}=M_{\text{s}}({\hat{x}}^{l+2})⊗{\hat{x}}^{l+2}$$

(12) $${\hat{x}}^{l+3}=x^{l+2}+x^{l+3}$$

In the equations above, *σ* is the activation function sigmod and MLP is multi-layer perceptron. $\text{Avg}P{\text{ool}}^{C\times 1\times 1}$ and $\text{Avg}{\text{Pool}}^{1\times H\times W}$ denote the global channel pooling and global spatial pooling, respectively. ${\text{MaxPool}}^{C\times 1\times 1}$ and ${\text{MaxPool}}^{1\times H\times W}$ denote maximum channel pooling and maximum spatial pooling, respectively. $Conv^{7\times 7}$ represents the convolution with a kernel size of 7×7. ⊗ denotes element multiplication.

### 3.3 Decoding module

To recover efficient semantic expressions, a decoding module containing up-sampling was employed to gradually recover the feature space information. The up-sampling was performed by the bilinear interpolation operator (26) with a scale factor of 2. Each decoding module consists of two consecutive series (convolution, batch normalization, Mish activation function), with the integral expression shown in Equation 13 and the Mish expression shown in Equation 14. All the convolutional layers in the decoding module have kernel size of 3×3 and padding size of 1. The number of channels is 512, 256, 128, and 64, respectively. The decoder can decompress the encoded medical image feature information and generate the corresponding dose distribution map.


(13) $$x^{l+4}={\left[\text{Mish}\{BN[{\text{conv}}^{C\times 3\times 3}({\hat{x}}^{l+3})]\}\right]}_{\times 2 up-sample}$$

(14) $$Mish(x)=x\cdot \tanh\left(\ln\left(1+e^{x}\right)\right)$$

For each decoding module, the scale resolution of the feature map increases by a factor of 2. The skip connection between encoding and decoding modules not only introduces spatial information but also alleviates the common gradient problem in deep learning.

### 3.4 Progressive refinement module

As shown in **Figure 2**, the progressive refinement (17) module contains four predictive branches *p_i_* to predict the dose distribution at different scale resolutions. Each prediction branch contains a generation module *G* to generate the dose distribution map $\varphi_{i}^{G}$ of size $n_{i}\times n_{i}$ . The generation module consists of two consecutive series (convolution, batch normalization, Mish activation function) with a scaling tuned convolution, as shown in Equation 15. To ensure constant output dimensionality, all convolutional layers have a convolutional kernel size of 3×3 and padding size of 1.


(15) $$\varphi_{i}^{G}=conv^{1\times 3\times 3}{\left[\text{Mish}\{BN[{\text{conv}}^{C\times 3\times 3}(x^{l+4})]\}\right]}_{\times 2}$$

The low-scale resolution dose distribution map $\varphi_{i}^{G}$ was fed to the higher-scale prediction branch after bilinear interpolation up-sampling operation with a scale factor of 2. Then, elementwise addition with the dose distribution map at high-scale resolution was used to obtain $\varphi_{i+1}^{G}$ , as shown in Equation 16.


(16) $$p_{i}=\{\begin{array}{ll} \varphi_{i}^{G}, & i=1\\ \varphi_{i+1}^{G}+U(\varphi_{i}^{G}), & i=2,3,4 \end{array}$$

Generally, low-scale resolution feature maps are easier to reconstruct than higher-scale resolution feature maps, and they pay more attention to detailed features. Thus, by utilizing the above-mentioned progressive refinement module, detailed information could be gradually added during the decoding process to generate more accurate dose distributions.

### 3.5 Multi-loss function fusion algorithm

To fit the dose distribution more accurately and accelerate the convergence of the model, we proposed the multi-loss function fusion algorithm (mean square error loss *L_m_*, planning target area loss *L_p_*, and rank loss *L_r_*) for weight optimization. Each prediction branch generated the loss score $L\left(p_{i},y_{i}\right)$ of the dose prediction *p_i_* compared to the ground truth *y_i_*, as shown in Equation 17.


(17) $$L\left(p_{i},y_{i}\right)=L_{m}\left(p_{i},y_{i}\right)+L_{p}\left(p_{i},y_{i}\right)+L_{r}\left(p_{i},y_{i}\right)$$

#### 3.5.1 Mean square error loss

In the dose prediction task, the mean square error (27) reflects the different degree of pixel points between images. The predicted dose *p_i_* is generally expected to be extremely close to the ground truth *y_i_*. Therefore, the mean square error was used as a loss function, as shown in Equation 18, where *m_i_* represents the number of dose pixels at level *i*.


(18) $$L_{m}=\frac{1}{2m_{i}}\|p_{i}-y_{i}{\|}_{2}^{2}$$

#### 3.5.2 Voxel-based loss

The voxel-based objective loss function (28) was designed based on the mean square error loss function, mainly using the PTV contour as a mask to generate the inner area *P_k_* and the outer area *N*. The internal dose distribution of the PTV is expected to be consistent with the gold standard since a too-high or too-low dose can result in an irreversible impact on patient treatment. Thus, for the inner area of the PTV, voxels that differ from the target value were penalized. Meanwhile, it is expected that the dose of the outer region of the PTV drops rapidly enough to avoid damage to normal tissue so that, for the outer area of the PTV, only the voxels higher than the target value were penalized. The weight coefficients were $\lambda_{k}=0.7\;\mu_{i}=0.3$ for the PTV region and the non-PTV region, respectively, and the loss function is shown in Equation 19. Here $p_{i}^{\alpha }$ and $p_{i}^{\beta }$ represent the predicted dose of the PTV and non-PTV region at layer *i*, respectively. $y_{i}^{\alpha }$ and $y_{i}^{\beta }$ represent the target dose of the PTV and non-PTV region at layer *i*, respectively. *P_i_* and *N_i_* represent the voxel number of the PTV and non-PTV region at layer *i*, respectively.


(19) $$L_{p}=\frac{\lambda_{k}\sum_{\alpha \in P_{k}}{\left(p_{i}^{\alpha }-y_{i}^{\alpha }\right)}^{2}}{2P_{i}}+\frac{\mu_{i}\sum_{\beta \in N}{\left[\max\left\{\left(p_{i}^{\beta }-y_{i}^{\beta }\;\right),0\right\}\right]}^{2}}{2{\text{N}}_{i}}$$

#### 3.5.3 Rank loss

The most direct assessment of the quality of the dose distribution is to measure the DVH, which usually uses the percentile of the dose distribution to determine the dose metric—for instance, *D*_98_ is the value of the 98th percentile in the dose distribution, which means that the sequential relation of dose values can reflect the dose distribution. Therefore, we proposed a rank-based loss function *L_r_* to ensure the order between the dose values in the dose prediction *p_i_* to be close to the real criteria *y_i_*. Firstly, the pixel values in *p_i_* and *y_i_* were vectorized and sorted in ascending order to get the pixel distribution $y_{i}^{*}$ of *y_i_* with the corresponding pixel index $\lambda_{index}$ , which was used to reconstruct the *p_i_* pixel vector in order to obtain $p_{i}^{*}$ . Secondly, the order of adjacent pixel metrics in $y_{i}^{*}$ and $p_{i}^{*}$ is shown in Equations 20 and 21, respectively, where $\sigma \in [1,m_{i}-1]$ .


(20) $$\rho (p_{i}^{*}(\sigma ),p_{i}^{*}(\sigma +1))=\frac{\exp(p_{i}^{*}(\sigma +1)-p_{i}^{*}(\sigma ))}{1+\exp(p_{i}^{*}(\sigma +1)-p_{i}^{*}(\sigma ))}$$

(21) $$\omega (y_{i}^{*}(\sigma ),y_{i}^{*}(\sigma +1))=\{\begin{array}{ll} 1, & y_{i}^{*}(\sigma +1)>y_{i}^{*}(\sigma )\\ 1/2, & y_{i}^{*}(\sigma +1)=y_{i}^{*}(\sigma )\\ 0, & y_{i}^{*}(\sigma +1)<y_{i}^{*}(\sigma ) \end{array}$$

Finally, the negative log-likelihood was used to measure the rank loss of *p_i_ versus y_i_*, as shown in Equation 22.


(22) $$L_{r}=-\frac{1}{2m_{i}}\sum_{j=1}^{m_{i}-1}\left[\begin{array}{l} \omega (y_{i}^{*}(\sigma ),y_{i}^{*}(\sigma +1))\cdot \log\rho (p_{i}^{*}(\sigma ),p_{i}^{*}(\sigma +1))+\\ \left(1-\omega (y_{i}^{*}(\sigma ),y_{i}^{*}(\sigma +1)))\cdot (1-\log\rho (p_{i}^{*}(\sigma ),p_{i}^{*}(\sigma +1))\right) \end{array}\right]$$

## 4 Result

### 4.1 Global dose prediction

As shown in **Table 1**, all four network models were trained using three different loss function algorithms. As can be seen in the table, DeepLab-V3-plus was slightly superior to PRT-Net in the MAE results obtained by training using the Lm&Lp algorithm, while in the remaining MSE, MAE, and RMSE indices, PRT-Net was the smallest of the four models. PRT-Net showed the least difference between the prediction and real data in the four models.

**Table 1:** MSE, MAE, and RMSE of the four models trained with three loss function algorithms.

	Metric	UNet	UNet ++	DeepLab −V3 −Plus	PRT-Net
***L_m_***	*MSE*	2.40 ± 1.85	1.97 ± 1.53	1.96 ± 1.87	**1.26 ± 1.08**
	*MAE*	0.72 ± 0.36	0.61 ± 0.34	0.62 ± 0.38	**0.52 ± 0.31**
	*RMSE*	1.35 ± 0.74	1.21 ± 0.69	1.16 ± 0.77	**0.95 ± 0.59**
***L_m_&L_p_***	*MSE*	2.33 ± 1.77	1.78 ± 1.57	2.13 ± 1.65	**1.12 ± 0.98**
	*MAE*	0.73 ± 0.44	0.55 ± 0.41	**0.55 ± 0.39**	0.57 ± 0.29
	*RMSE*	1.33 ± 0.76	1.19 ± 0.58	1.23 ± 0.86	**0.88 ± 0.61**
***L_m_&L_p_&L_r_***	*MSE*	2.45 ± 1.83	1.88 ± 1.41	1.76 ± 1.77	**1.31 ± 1.12**
	*MAE*	0.69 ± 0.33	0.57 ± 0.39	0.61 ± 0.44	**0.49 ± 0.33**
	*RMSE*	1.30 ± 0.72	1.21 ± 0.66	1.09 ± 0.59	**0.92 ± 0.71**

The smallest value in each row is highlighted with bold font. Results are given as mean ± standard deviation.

We divided the remaining results into two parts, showing the comparison between the four network models and the comparison between the three loss function models.

#### 4.1.1 Comparison between the four network models

**Figure 3:**
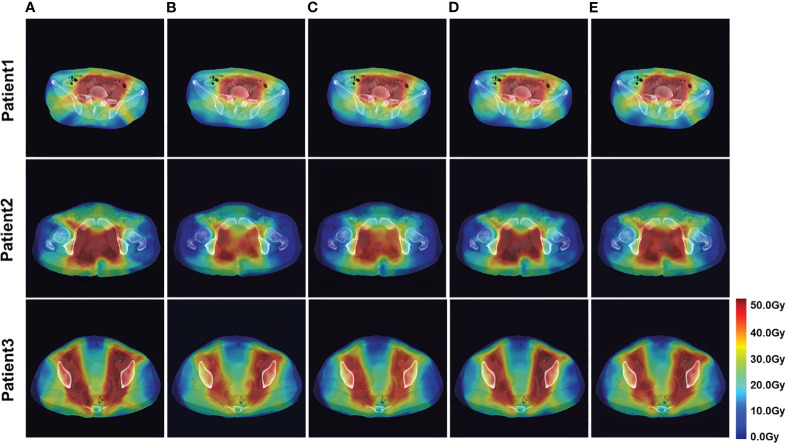
Example of dose predictions. The real dose distribution and the dose distribution predicted by the four models in color wash are included. **(A)** Label, **(B)** U-Net, **(C)** U-Net++, **(D)** DeepLab-V3-PLUS, and **(E)** PRT-Net.

**Figure 4:**
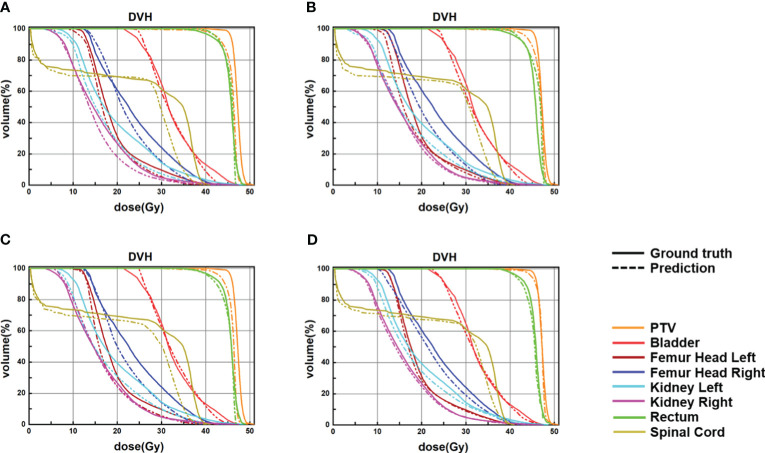
Samples of DVHs derived from the real dose distributions and dose predictions from U-Net, U-Net++, DeepLab-V3-plus, and the proposed PRT-Net. The solid lines are DVHs of the real dose distribution, and the dotted lines are DVHs of the different models’ dose predictions. **(A)** U-Net, **(B)** U-Net++, **(C)** DeepLab-V3-PLUS, and **(D)** PRT-Net.

**Figure 5:**
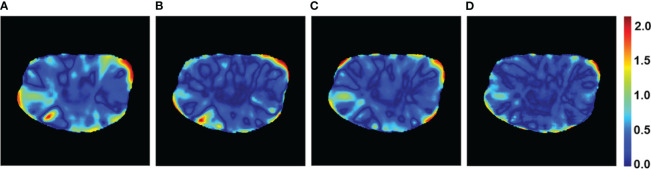
Gamma passing rate with 3%/2 mm criteria of a patient case for the four models. **(A)** U-Net, **(B)** U-Net++, **(C)** DeepLab-V3-PLUS, and **(D)** PRT-Net.

The four network models were trained separately using the Lm loss function algorithm, and the results of each are shown below. **Table 2** demonstrates the absolute differences between the dosimetric parameters predicted by the four neural network models and the real ones. As can be seen in the table, PRT-Net achieved optimal results for several metrics (PTV D2, bladder V30, V40, V45, rectum V30, V40, left femoral head, and right femoral head V30 had the smallest absolute errors among the four models). U-net++ also showed good results on several indicators (D95 and D98 for PTV and Dmax for the spinal cord). **Figure 3** shows the dose distribution of three patients in the test cohort, including the real dose distribution and predicted outcomes in four models. PRT-Net is the closest to the real dose distribution in predicting the high dose range and the low dose range by comparing the predictions of the four models. **Figure 4** shows the DVH curves predicted by the four network models. The OARs and PTV curves show that the DVH curves predicted by PRT-Net are the closest to the real curve in the four models, especially the PTV curves. **Figure 5** shows the results of the 2D gamma analysis with 3%/2 mm criteria. This suggests that the U-net model is relatively poor at predicting VMAT doses, while PRT-Net has the highest gamma pass rate and the closest approximation to true dose distribution.

**Table 2:** The differences in the quantitative dosimetric metrics between real dose distribution and the four models’ dose prediction.

ROIs	Metric	UNet	UNet++	DeepLab – V3 – Plus	PRT – Net
**PTV**	*D*_95(_ *_Gy_*_)_	2.58 ± 0.57	**0.59 ± 0.51**	1.44 ± 0.37	1.11 ± 0.29
	*D*_98(_ *_Gy_*_)_	3.98 ± 1.03	**1.67 ± 0.72**	2.43 ± 0.48	1.96 ± 0.52
	*D*_2(_ *_Gy_*_)_	1.17 ± 0.43	0.89 ± 0.52	0.80 ± 0.42	**0.75 ± 0.40**
**Bladder**	*V*_30(%)_	4.65 ± 3.42	2.89 ± 2.29	2.65 ± 2.72	**2.64 ± 1.54**
	*V*_40(%)_	6.18 ± 2.73	3.85 ± 3.66	3.19 ± 2.40	**2.95 ± 1.68**
	*V*_45(%)_	8.74 ± 5.57	5.67 ± 4.99	5.83 ± 3.83	**4.33 ± 2.49**
**Rectum**	*V*_30(%)_	1.55 ± 1.44	1.79 ± 1.77	2.95 ± 3.79	**1.45 ± 1.37**
	*V*_40(%)_	6.04 ± 4.02	6.25 ± 4.40	6.40 ± 4.51	**3.64 ± 2.83**
**Femoral left**	*V*_30(%)_	2.74 ± 1.44	1.94 ± 2.27	2.50 ± 1.72	**1.56 ± 2.05**
**Femoral right**	*V*_30(%)_	2.16 ± 1.42	2.33 ± 1.39	1.75 ± 1.48	**1.33 ± 1.25**
**Spinal cord**	*D_max_*_(_ *_Gy_*_)_	1.91 ± 1.89	**1.56 ± 1.55**	1.78 ± 1.61	1.67 ± 1.38

The smallest value in each row is highlighted in bold font. Results are given as mean ± standard deviation.

#### 4.1.2 Comparison between the three loss function models

**Figure 6:**
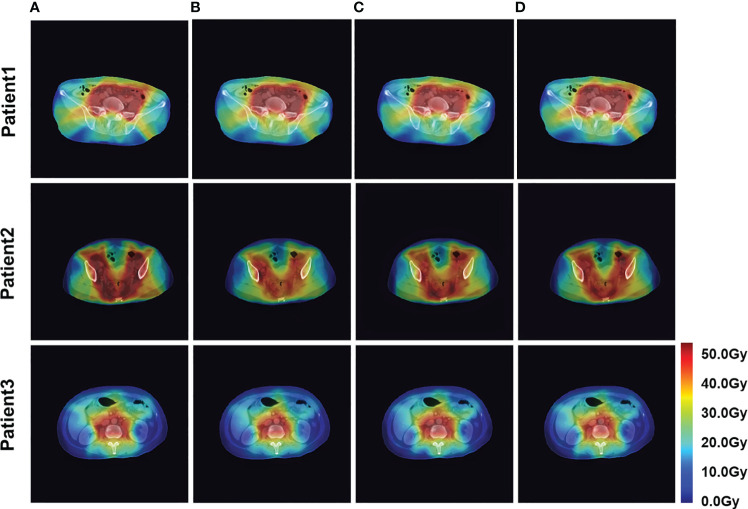
Example dose predictions. The real dose distribution and the dose distribution predicted by the PRT-Net model trained with three loss function algorithms are included. **(A)** Label, **(B)** Lm, **(C)** Lm&Lp, and **(D)** Lm&Lp&Lr.

**Figure 7:**
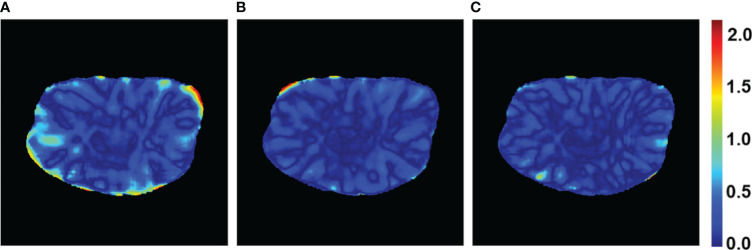
Gamma passing rate with 3%/2 mm criteria of a patient case for the PRT-Net model trained with three loss function algorithms. **(A)** Lm, **(B)** Lm&Lp, and **(C)** Lm&Lp&Lr.

The PRT-Net model was trained using three loss function algorithms, Lm, Lm&Lp, and Lm&Lp&Lr, respectively, and the results are shown below. **Table 3** shows the absolute dose difference between the predicted dose distribution and the real dose distribution for dose indicators. As can be seen in the table, for PTV D2, D98, D95, bladder V30, V40, left femoral head, and right femoral head V30, the Lm&Lp&Lr algorithm model had the least error and the best prediction. The results show that using the Lm&Lp&Lr algorithm as a loss function can reduce the prediction error to some extent. **Figure 6** shows the dose distribution of a patient. From the figure, the model is better able to characterize the dose distribution after fusion of the Lm&Lp&Lr algorithm than using the Lm algorithm alone as a loss function. As shown in **Figure 7**, in the 2D gamma analysis with 3%/2 mm criteria, the bright color range of the Lm&Lp&Lr algorithm model is the smallest, especially the range of the low dose region near the body. It indicates that using the Lm&Lp&Lr algorithm as a loss function is also able to predict the low dose region more accurately to some extent, and its predicted dose distribution is closest to the real dose distribution.

**Table 3:** The differences of the quantitative dosimetric metrics between real dose distribution and the prediction from the PRT-Net model trained with three loss function algorithms.

ROIs	Metric	*L_m_*	*L_m_* &*L_p_*	*L_m_* &*L_p_* &*L_r_*
**PTV**	*D*_95(_ *_Gy_*_)_	1.11 ± 0.29	0.79 ± 0.39	**0.57 ± 0.44**
	*D*_98(_ *_Gy_*_)_	1.96 ± 0.52	1.59 ± 0.57	**0.95 ± 0.86**
	*D*_2(_ *_Gy_*_)_	0.75 ± 0.40	0.72 ± 0.38	**0.59 ± 0.28**
**Bladder**	*V*_30(%)_	2.64 ± 1.54	4.49 ± 4.17	**2.45 ± 2.11**
	*V*_40(%)_	2.95 ± 1.68	3.84 ± 3.33	**2.77 ± 2.02**
	*V*_45(%)_	**4.33 ± 2.49**	4.92 ± 4.20	4.40 ± 4.14
**Rectum**	*V*_30(%)_	1.45 ± 1.37	**1.06 ± 0.88**	1.12 ± 0.85
	*V*_40(%)_	3.64 ± 2.83	**2.60 ± 1.98**	3.05 ± 2.77
**Femoral left**	*V*_30(%)_	1.56 ± 2.05	1.58 ± 1.73	**1.38 ± 1.23**
**Femoral right**	*V*_30(%)_	1.33 ± 1.25	1.45 ± 1.60	**1.11 ± 1.00**
**Spinal cord**	*D_max_*_(_ *_Gy_*_)_	1.67 ± 1.38	**0.88 ± 0.73**	1.69 ± 0.93

The smallest value in each row is highlighted with bold font. Results are given as mean ± standard deviation.

### 4.2 Dose prediction in the low dose region

To determine the appropriate location of the ovary during ovarian transposition, it is necessary to focus on the prediction of low dose regions in this study. To demonstrate that image alignment has a small effect on the low dose region of interest in this study, it is ensured that the constructed dose prediction model can accurately predict the low dose distribution. The dice similarity coefficient (DSC), Hausdorff distance (HD), and Jaccard coefficient after rigid alignment were calculated for isodose volumes of 4, 10, 15, and 20 Gy, and the results are shown in the **Table 4** (training by Lm loss). The DSC for 4, 10, 15, and 20 isodose are all higher than 0.97, the Hausdorff distances are all between 1.8 and 2.0 cm, and the Jaccard coefficients are all higher than 0.9. This indicates that the low isodose volume on the preoperative CT obtained by the rigid alignment is very close to that of the planning CT.

**Table 4:** DSC, Hausdorff distance, and Jaccard coefficient for low-isodose volume after rigid alignment.

ROIs	DSC	Hausdorff distance (cm)	Jaccard coefficient
4 Gy	0.974 ± 0.041	1.940 ± 1.707	0.948 ± 0.054
10 Gy	0.973 ± 0.082	1.808 ± 1.354	0.951 ± 0.047
15 Gy	0.963 ± 0.076	2.006 ± 1.323	0.951 ± 0.041
20 Gy	0.966 ± 0.064	1.898 ± 1.246	0.942 ± 0.040

The dose predictions in the low dose region of each model are shown below. The mean DSC of 4, 10, 15, and 20 Gy for the 13 test set data are shown in **Table 5** (training by Lm loss) and **Table 6**. It can be seen that, among the four models, the DSC of DeepLab-V3-plus is slightly higher than that of PRT-Net for the 10-Gy isodose, and the DSC of PRT-Net is the highest among the four models for the remaining metrics. The DSC values of PRT-Net are all higher than 0.96. As shown in **Table 6**, although Lm&Lp&Lr loss did not produce the best DSC metric for the 4-Gy isodose line, the 95% Hausdorff distance (HD95) is the smallest. In addition, compared to Lm loss and Lm&Lp loss, Lm&Lp&Lr loss still achieved the best performance in the prediction of 4- to 20-Gy isodose lines. Thus, Lm&Lp&Lr loss is more appropriate to guide the low dose region to move the ovary to.

**Table 5:** Iso-dose dice similarity coefficient (DSC) between clinical and predicted isodose volumes for the four models.

	UNet	UNet++	DeepLab – V3 – Plus	PRT – Net
**4 *Gy***	0.920 ± 0.120	0.957 ± 0.072	0.968 ± 0.114	**0.974 ± 0.041**
**10 *Gy***	0.933 ± 0.071	0.971 ± 0.052	**0.976 ± 0.091**	0.973 ± 0.082
**15 *Gy***	0.955 ± 0.118	0.954 ± 0.073	0.961 ± 0.080	**0.963 ± 0.076**
**20 *Gy***	0.949 ± 0.046	0.961 ± 0.105	0.944 ± 0.055	**0.966 ± 0.064**

The best result in each row is highlighted in bold font.

**Table 6:** Iso-dose dice similarity coefficient (DSC) and 95% Hausdorff distance (HD95) between clinical and predicted isodose volumes for the PRT-Net model trained with three loss function algorithms.

	*L_m_*		*L_m_* &*L_p_*		*L_m_* &*L_p_* &*L_r_*	
	DSC	HD95	DSC	HD95	DSC	HD95
**4 *Gy***	**0.974 ± 0.04**	1.103 ± 0.99	0.966 ± 0.05	1.215 ± 1.01	0.968 ± 0.114	**1.032 ± 0.87**
**10 *Gy***	0.973 ± 0.08	**1.097 ± 1.11**	**0.976 ± 0.03**	1.133 ± 0.99	**0.976 ± 0.093**	1.152 ± 1.31
**15 *Gy***	0.963 ± 0.07	1.336 ± 1.21	0.964 ± 0.09	**1.227 ± 1.04**	**0.972 ± 0.062**	1.313 ± 1.19
**20 *Gy***	0.966 ± 0.06	1.297 ± 0.83	0.961 ± 0.10	1.306 ± 1.08	**0.969 ± 0.082**	**1.282 ± 1.29**

The best result in each row is highlighted in bold font.

**Figure 8:**
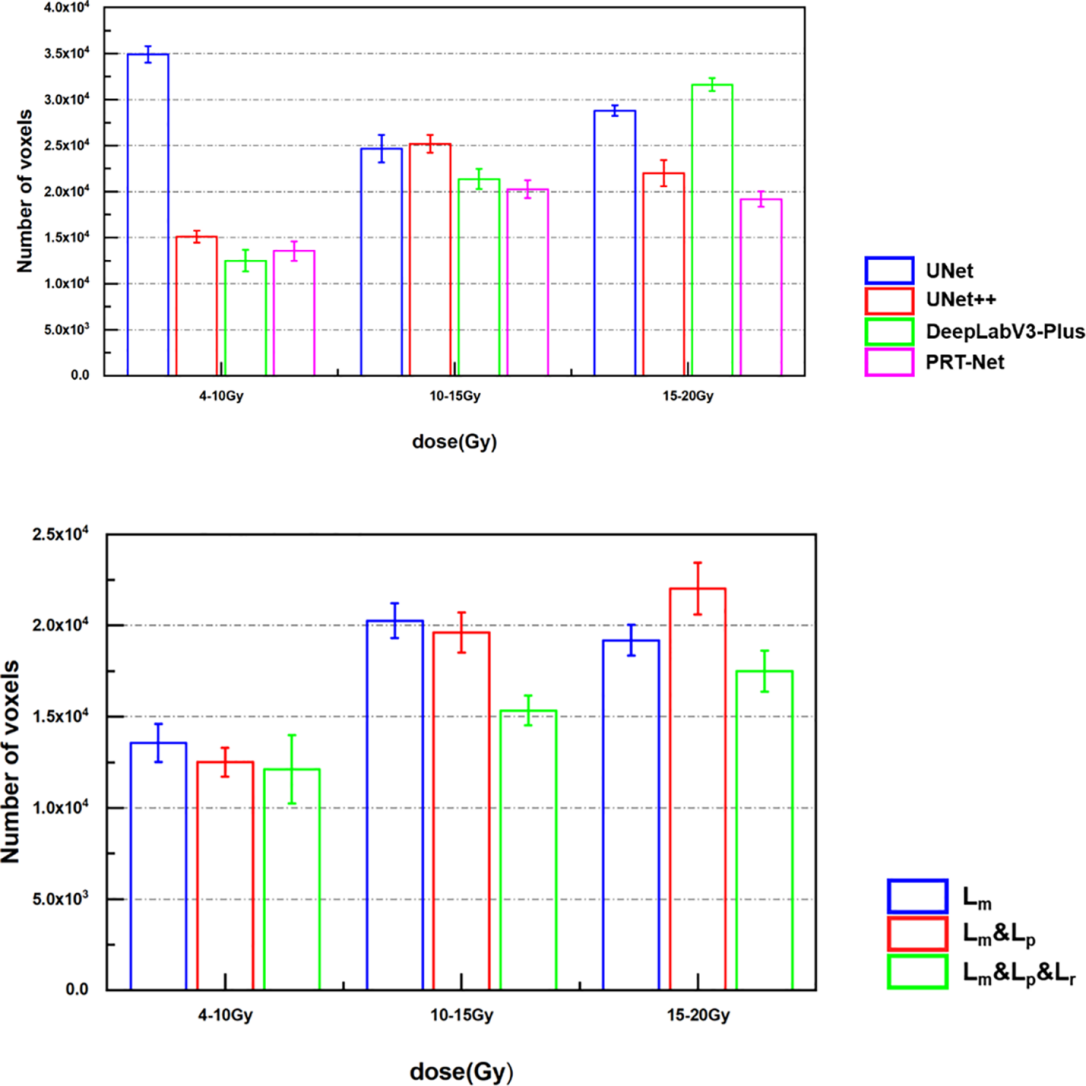
Difference between the real voxel number and the predicted number from the models.

In order to quantitatively evaluate the prediction accuracy in the low dose range, we counted the number of voxels predicted by the models for 4–10 Gy, 10–15 Gy, and 15–20 Gy dose region and the number of voxels in the real dose distribution for the corresponding dose regions to find the average absolute difference between the predicted and real dose. From **Figure 8**, we can see that among the four models, 4–10 Gy voxel number predicted by DeepLab-V3-plus had the smallest error, which was slightly better than that predicted by PRT-Net. The prediction by PRT-Net generated the smallest error of voxel numbers for 10–15 and 15–20 Gy dose regions among the four models. Overall, PRT-Net was better at predicting low dose regions than the other three models. After incorporating the Lm&Lp&Lr algorithm, the errors in number of voxel predicted by the model decreased for 4–10-, 10–15-, and 15–20-Gy dose regions, indicating a further improvement in the model’s ability to predict low dose regions.

### 4.3 Application to clinical case

**Figure 9:**
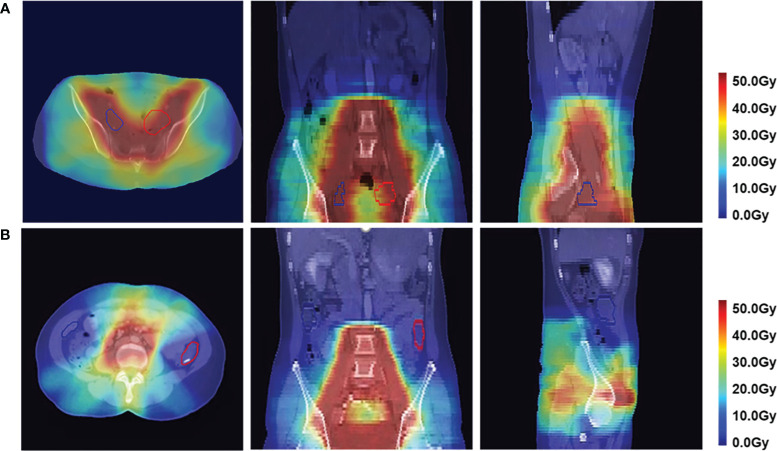
Dose distribution in axial, coronal, and sagittal views in color wash in a patient. The red contour is the left ovary, and the blue contour is the right ovary. **(A)** Predicted postoperative dose distribution overlaid on preoperative CT. **(B)** Postoperative dose distribution.

**Figure 10:**
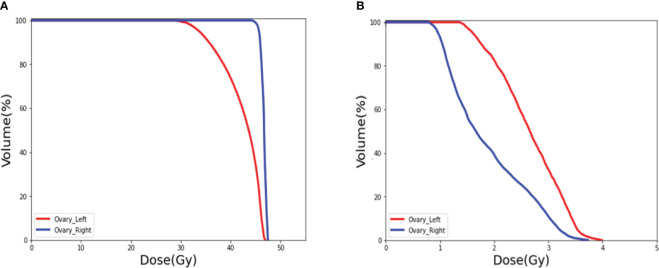
DVH of the ovaries. **(A)** Prediction of the ovary dose on preoperative CT. **(B)** Postoperative ovarian dose after ovarian transposition using our method as guidance.

To validate the clinical feasibility of our proposed method, the preoperative CT of a patient who will undergo ovarian transposition and receive postoperative radiotherapy at our hospital was selected as input into our dose prediction model. The predicted dose distribution combined with the preoperative CT was provided to the surgeon as shown in **Figure 9A**. The approximate range of the low dose within which we recommend moving the ovaries to can be seen in the figure (blue area in the figure). Based on this result, the surgeon moved the ovaries to within our recommended safe dose range during the ovarian transposition surgery. The postoperative radiotherapy plan based on the postoperative CT is shown in **Figure 9B**. Additionally, the DVH of the ovaries on the preoperative CT and postoperative CT were compared to determine whether the dose to the ovaries was reduced and the clinical feasibility of our proposed method, as shown in **Figure 10**.

**Figure 10A** shows the DVH of the ovary when ovarian transposition surgery has not yet been performed. As can be seen in the figure, without ovarian transposition, the ovaries will be treated with postoperative radiotherapy at doses as high as the prescribed dose of 45 Gy or even higher. **Figure 10B** shows the DVH of the ovary in postoperative radiotherapy after ovarian transposition surgery using our proposed method as guidance. By moving the ovaries to the predicted safe dose area, the dose to the ovaries in postoperative radiotherapy will be greatly reduced to within the safety limit of 4 Gy.

## 5 Discussion

In this study, we proposed a novel progressive refinement attention model PRT-Net for predicting the location of transposed ovary before surgery to assist surgeons in their clinical work. Compared to U-net, U-net ++, and DeepLab-V3-plus, three widely used network models for dose prediction studies, PRT-Net has enhanced feature extraction and learning capabilities. In the high dose region near PTV, PRT-Net had a prediction error of 1.11 ± 0.29 Gy for PTV D95 compared to 2.58 ± 0.57 Gy for classic U-Net and 1.44 ± 0.37 Gy for DeepLab-V3-plus. The accuracy of the PRT-Net model in predicting high dose is improved. In the low dose range, the DSC of PRT-Net was 0.974 ± 0.04 for 4 Gy isodose, which was also better than that of U-net, U-net++, and DeepLab-V3-plus (0.920 ± 0.12, 0.957 ± 0.07, and 0.968 ± 0.11, respectively), and the dose predicted by PRT-Net was closer to the real data. Combining the PRT-Net with the Lm&Lp&Lr algorithm significantly improved the model’s ability to predict dose distribution by more accurately characterizing the relationship between contour location and dose, reducing the predicted PTV D95 error from 1.11 ± 0.29 to 0.57 ± 0.44 Gy.

The PRT-Net model uses an enhanced self-attentive module to fit the inter-correlation information between different features. It uses a convolutional attention mechanism to infer the weight concentration region in the channel and spatial dimensions and adaptively optimize local features. Moreover, the model can generate dose predictions at four different resolutions during a forward propagation by the refinement module and improve the dose prediction results by gradually fitting the high resolution at low resolution. A multi-loss function fusion algorithm was proposed to improve the prediction results in different loss dimensions. A deep supervised training algorithm was deployed to jointly optimize three different loss functions: mean square error loss, planning target region loss, and sequence loss.

The reason for using preoperative CT rather than planning CT as model input in this study is based on the anatomy. In previous dose prediction studies, researchers have used the planning CT as the input to the model. However, for patients undergoing radical hysterectomy and postoperative radiotherapy, a planning CT will show that the surgery has been completed and the ovaries are located in a way that cannot be easily changed. Therefore, it is illogical to use planning CT for dose prediction to determine the safe location of the ovaries. To predict the safe location of the ovary prior to surgery, preoperative CT must be used as the model input.

Since no other deep learning-based dose prediction studies are using preoperative CT as model input, it is difficult to compare the dose prediction results of this study with other dose prediction studies in the literature. The reason why other studies did not use preoperative CT for dose prediction was mainly because the location and size of OARs on preoperative CT and planning CT differ depending on the drugs used during chemotherapy and radical hysterectomy. The deformation of the OARs resulted in a significant variation of dose distribution in the high dose regions; thus, the results predicted by the model in the high dose regions (e.g., dosimetric parameters for rectum and bladder) cannot be applied clinically. The DVH curve in **Figure 4** shows a particularly high dose in the rectum, mainly due to the deviations in the position of the rectum on preoperative and planning CT. The rectum on the preoperative CT was just on the location of the PTV in the planning CT, which resulted in the dose level to the rectum on the preoperative CT being close to the PTV. However, our study found that the location of isodose lines in low dose areas such as 4 and 10 Gy was minimally affected by the deformation of OARs. As shown in **Table 4**, the variation of the low isodose volume after rigid image alignment is very small. For this reason, this study was able to accurately predict the dose distribution in the low dose region by preoperative CT, thus predicting the safe location of the transposed ovary. Therefore, the deformation of OARs has a significantly negative impact on predicting parameters associated with OARs in the high dose region but has a little effect on the dose distribution in the low dose region of interest in this study.

In addition, even if the preoperative CT dose distribution differs slightly from the planning CT dose distribution due to factors such as the deformation of OARs and the difference in treatment couch, the surgeon and physicists can further fine-tune the dose of the ovary to bring it below the safe dose limit by the subsequent design of the RT plan. The position of the 4-Gy isodose line shown on the dose distribution is not always suitable for the fixation and placement of the transposed ovary due to procedural difficulties and other factors. Therefore, the surgeon can also choose to transpose ovaries to 5–10-Gy areas and adjust the ovarian dose through subsequent RT treatment plans to ensure that the dose is below the safe limit. Using the method proposed in this study can prevent surgeons from mistakenly transposing ovaries into the 20- and 30-Gy high dose regions without knowing the dose distribution. In such cases, it is difficult to control the dose of the ovary below the safe limit in subsequent treatment planning, which may either lead to insufficient coverage in PTV or sacrifice of the protection of the ovary. The dose distribution generated by artificial intelligence also provides a visual reference for the surgeon preoperatively, allowing them to specify the appropriate ovarian transplant location and reduce the procedure’s difficulty and risk. Note that, in this study, the ovaries were excluded from the structures, and the ovarian objective function was not included in the plan optimization, so the AI model did not learn the location information of ovarian displacement selected by the surgeons. Therefore, the method proposed in this study can help surgeons quickly determine the approximate range of safe ovarian locations, significantly reducing the risk of surgeons leaving the ovaries in the possible high dose area.

Differently from previous research, our research focuses on using artificial intelligence to solve a specific challenge in practical clinical work, which is to determine the location for ovarian transposition. The main focus of research for utilizing neural network in dose prediction was to improve the accuracy of network model prediction, making the prediction closer to the clinical plan, or to automatically generate a deliverable plan through artificial intelligence (29–31). Wang et al. (17) proposed a novel progressive refinement U-Net with rank loss to predict the VMAT dose of prostate cancer end-to-end, using the multi-task learning training strategy to optimize the output details, which had a significant improvement compared to traditional U-net models. Sun et al. (28) proposed a voxel optimization strategy based on the U-net algorithm, which used the PTV binary contour as a mask to generate the inner and outer regions, then utilized these two regions to optimize the treatment plan, and applied a “hybrid” optimization strategy to generate personalized radiotherapy plan. In contrast, our research was aimed at assisting clinicians to perform their tasks more efficiently and accurately by introducing deep learning methods to protect the ovaries of young cervical cancer patients. In this work, U-net had the weakest VMAT dose prediction ability among the four models, especially for the low dose region, and these results were similar to those of Wang et al. (17). Even compared with U-net++ and DeepLab-V3-Plus, the proposed PRT-Net has better dose prediction ability. Currently, although commercial TPS software is available to provide commercial dose prediction functions (e.g., Rapidplan knowledge-based planning), Rapidplan mainly predicts DVH to obtain an objective function to optimize the plan (32, 33). It is based on postoperative planning CT for dose prediction, but the results of dose prediction using preoperative CT are not clear for the time being. The surgeon needs to combine the complete dose distribution with the preoperative CT to accurately determine the location of the transposed ovary. Therefore, we constructed a progressive refinement module to improve the accuracy of predicting low dose regions based on preoperative CT and thus more accurately predict the location of safe ovarian transposition. The network is more applicable to assist in the clinical performance of ovarian transposition.

There are also some limitations of this study. First, in actual clinical practice, ovarian survival is closely related not only to the dose in radiotherapy but also to the age of the patient and the drugs used in the concurrent chemotherapy (2, 34). However, for cervical cancer patients who need to maintain ovarian function, age is the determining factor. Moreover, to ensure the efficacy of treatment, it is difficult to change the chemotherapy schedule. Thus, only the dose of radiotherapy is easier to limit and control (8). This study provides technical guidance for current ovarian function protection in terms of radiation doses. If we want to further improve the ovarian survival rate, several factors such as the use of chemotherapy drugs need to be considered. Second, in actual clinical diagnosis, PTV is usually delineated based on multiple adjacent slices along the cranial–caudal direction (Z-axis). However, the 2D convolutional kernels approach ignores the context along the Z-axis, resulting in losing spatial congruence information. Specifically, the single slice or three consecutive slices cropped from 3D volumetric images were fed to the 2D neural networks, and the 3D dose volume was generated by simply stacking the 2D dose map. Although using adjacent slices, it still cannot fully exploit the spatial information in three dimensions, which may bias the prediction results. Furthermore, rigid registration was utilized in this study due to the unpredictable deformation of structures during hysterectomy, although rigid registration was sufficient to achieve the goal of this study, which is to predict the low dose regions, as proven by the high DSC and HD95. The possible reason is that the low dose area is large, and the most important factors affecting its volume and location are the shape and location of the PTV. Since the shape and position of PTV on preoperative CT and postoperative CT of cervical cancer are relatively stable and unchanged, the method using rigid alignment is able to let neural network predict the low dose area. Future work may include training of neural network on learning of preoperative CT to postoperative CT registration for a more robust predicted dose distribution. Finally, the transformer-based neural network architecture requires large data sets to eliminate overfitting. However, so far, the local institution has only 104 cases available for dose prediction, which may lead to prediction bias. Maybe the more optimized transformer algorithms, such as the axial self-attention model (35) or gated-attention-model (36), could alleviate the overfitting. Future work includes implementing our model to 3D dose prediction, applying our method to more clinical cases, and exploring the possibility of improving ovarian survival in young cervical cancer patients by combining various factors such as chemotherapy. In addition, we will consider applying our model to other clinical work to assist clinicians with risk assessment and decision analysis, making the clinical work less difficult and more efficient.

## 6 Conclusion

In this work, we propose PRT-Net based on reinforced self-attentive architecture, which deployed a multi-loss function fusion algorithm to train the progressive refinement module to fit the dose prediction distribution. It is challenging to predict the dose distribution by preoperative CT due to factors such as the deformation of OARs, which has higher requirements for neural network model algorithms. Low dose regions were successfully predicted based on the patient’s preoperative CT. The results were applied to ovarian transposition to reduce the risk of ovaries in the high dose region.

## Data availability statement

The original contributions presented in the study are included in the article/supplementary material. Further inquiries can be directed to the corresponding authors.

## Ethics statement

Written informed consent was obtained from the individual(s) for the publication of any potentially identifiable images or data included in this article.

## Author contributions

SL: Writing – original draft, Writing – review & editing, Conceptualization, Data curation, Formal analysis, Investigation, Methodology, Resources, Software, Supervision, Validation, Visualization. YD: Writing – original draft, Writing – review & editing, Data curation, Investigation, Project administration. CW: Writing – original draft, Writing – review & editing, Data curation, Methodology, Software, Supervision. YH: Supervision, Writing – review & editing. ZY: Validation, Writing – review & editing. HQ: Validation, Writing – review & editing. CM: Writing – review & editing. BZ: Project administration, Visualization, Writing – original draft, Writing – review & editing. XX: Data curation, Formal analysis, Methodology, Project administration, Software, Supervision, Writing – original draft, Writing – review & editing. WW: Conceptualization, Data curation, Funding acquisition, Project administration, Resources, Supervision, Validation, Visualization, Writing – original draft, Writing – review & editing. XW: Formal analysis, Supervision, Validation, Visualization, Writing – review & editing.
